# Heat-Killed *Fusobacterium nucleatum* Triggers Varying Heme-Related Inflammatory and Stress Responses Depending on Primary Human Respiratory Epithelial Cell Type

**DOI:** 10.3390/molecules25173839

**Published:** 2020-08-24

**Authors:** Ryo Koike, Marni E. Cueno, Keiko Nodomi, Muneaki Tamura, Noriaki Kamio, Hajime Tanaka, Ai Kotani, Kenichi Imai

**Affiliations:** 1 Division of Oral Structural and Functional Biology, Nihon University Graduate School of Dentistry, Tokyo 101-8310, Japan; koike.ryou@g.nihon-u.ac.jp; 2 Department of Microbiology, Nihon University School of Dentistry, Tokyo 101-8310, Japan; nodomi.keiko@nihon-u.ac.jp (K.N.); tamura.muneaki@nihon-u.ac.jp (M.T.); kamio.noriaki@nihon-u.ac.jp (N.K.); tanaka.hajime@nihon-u.ac.jp (H.T.); 3 Department of Hematological Malignancy, Institute of Medical Science, Tokai University, Kanagawa 259-1193, Japan; aikotani@k-lab.jp

**Keywords:** *Fusobacterium nucleatum*, fusobacterial adhesin, heat-killed, heme, inflammatory response, virulence potential

## Abstract

*Fusobacterium nucleatum* (Fn) is generally an opportunistic oral pathogen that adheres to mammalian mucosal sites, triggering a host inflammatory response. In general, Fn is normally found within the human oral cavity; however, it was previously reported that Fn is a risk factor for certain respiratory diseases. Surprisingly, this was never fully elucidated. Here, we investigated the virulence potential of heat-killed Fn on primary human tracheal, bronchial, and alveolar epithelial cells. In this study, we measured the secretion of inflammatory- (IL-8 and IL-6), stress- (total heme and hydrogen peroxide), and cell death-related (caspase-1 and caspase-3) signals. We established that the inflammatory response mechanism varies in each epithelial cell type: (1) along tracheal cells, possible Fn adherence would trigger increased heme secretion and regulated inflammatory response; (2) along bronchial cells, potential Fn adherence would simultaneously initiate an increase in secreted H_2_O_2_ and inflammatory response (ascribable to decreased secreted heme amounts); and (3) along alveolar cells, putative Fn adherence would instigate the increased secretion of inflammatory responses attributable to a decrease in secreted heme levels. Moreover, regardless of the epithelial cell-specific inflammatory mechanism, we believe these are putative, not harmful. Taken together, we propose that any potential Fn-driven inflammation along the respiratory tract would be initiated by differing epithelial cell-specific inflammatory mechanisms that are collectively dependent on secreted heme.

## 1. Introduction

*Fusobacterium* species are Gram-negative, anaerobic bacterial species that are commonly found in mammalian mucosal sites and, similarly, the presence of these species along both healthy tissues and disease sites insinuate that these species are opportunistic pathogens [1]. Among the various *Fusobacterium* species known, *Fusobacterium nucleatum* (Fn) is the most abundant in the human oral cavity and serves as a key member of the human oral biofilm [1,2]. In addition, Fn has been widely associated with periodontal disease and implicated in several systemic diseases, including preterm birth and colorectal cancer [2,3,4,5,6]. Moreover, Fn is a potent stimulator of inflammatory cytokines resulting to exacerbated inflammation [7] which, consequently, is one of the traits that make Fn a major pathogen [8]. Taken together, this would imply that Fn-driven inflammation plays a role in disease induction. Surprisingly, respiratory diseases have also been considered a risk factor for Fn [9,10] which would suggest that Fn-driven inflammation may also contribute to respiratory disease development.

Another important role of Fn is that it bridges organisms together since it forms polymicrobial interactions between species [3], owing to several fusobacterial adhesins [2]. At present, there are five identified fusobacterial adhesins: Aid1, CmpA, Fap2, FomA, and RadD. Aid1 is an arginine-inhibitable adhesin found to play a role in facilitating the interaction between Fn and oral streptococci [11]. CmpA is also an arginine-inhibitable adhesin involved in the interaction between Fn and *Streptococcus gordonii* [12]. Fap2 is a galactose-inhibitable adhesin associated with the interaction between Fn and *Porphyromonas gingivalis* [13]. FomA is putatively an arginine-inhibitable adhesin capable of binding to varying bacterial species and human epithelial cells [14]. Bacterial adherence is considered an important virulence factor [15]; however, the virulence potential and mechanism of Fn adherence (mainly attributable to fusobacterial adhesins) is not fully understood [2]. Considering Fn adherence is a possible virulence factor and Fn induces inflammation, we hypothesize that Fn adherence (possibly via fusobacterial adhesins) may contribute to Fn-driven inflammation in respiratory tissues, making it a risk factor for respiratory disease development. A better understanding of the potential association between Fn-related adhesion and Fn-driven inflammation could shed light on the virulence mechanism of Fn and, equally importantly, lead to novel therapeutic strategies that could inhibit Fn-related adhesion on host tissues.

## 2. Results

### 2.1. Secreted IL-8 and IL-6 Are Affected by Bacterial Incubation Time and Cell Count

**Figure 1:**
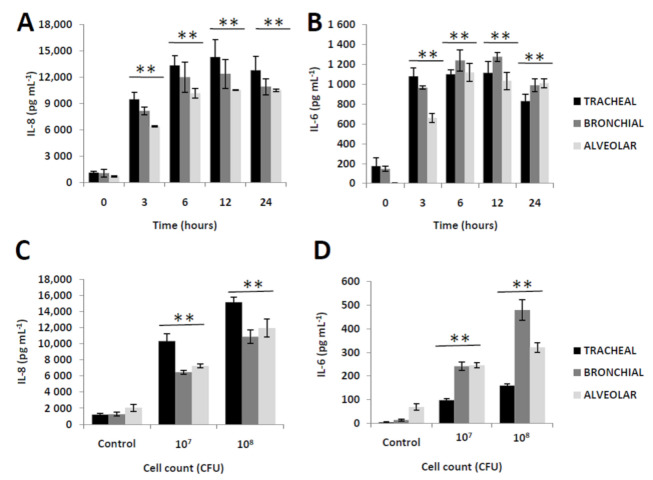
Inflammatory responses associated to heat-killed *Fusobacterium nucleatum* are affected by bacterial incubation time and cell count. Assay measurements of secreted IL-8 and IL-6 from human primary tracheal, bronchial, and alveolar epithelial cells incubated with heat-killed *F*. *nucleatum* (1.0 × 10^8^ CFU mL^−1^) are specified. (**A**) Secreted IL-8 and (**B**) IL-6 amounts at varying incubation times are shown. (**C**) Secreted IL-8 and (**D**) IL-6 under the same infection time point (12 h) and different bacterial cell concentration are indicated. The results presented are the mean ± SD utilizing two replicates of three independent samples. Statistical analyses were performed using an Andersen–Darling normality test to check whether the values obtained were normalized (*p* > 0.05) and, if passed, matched-pair *t*-test (** *p* < 0.01).

Foreign compounds and pathogens have been known to stimulate the secretion of inflammatory cytokines (such as IL-8 and IL-6) [16,17,18,19] which, in turn, is affected by the time and robustness of epithelial cell activation leading to the release of high levels of proinflammatory cytokines [20]. To determine whether extracellular inflammatory activity from human respiratory epithelial cells is affected by bacterial incubation time and cell count, secreted IL-8 and IL-6 amounts were measured. As seen in Figure 1A (IL-8) and 1B (IL-6), the presence of bacterial cells regardless of incubation time induced both IL-8 and IL-6 extracellular secretion at varying levels depending on the type of respiratory epithelial cell. More specifically, the tracheal extracellular IL-8 amount was highest at 12h post-incubation, whereas both extracellular bronchial and alveolar IL-6 amounts were highest at 6h post-incubation. Similarly, we observed that increasing bacterial cell number stimulates increasing secreted IL-8 (Figure 1C) and IL-6 (Figure 1D) amounts in all epithelial cell types. These results would insinuate that for both IL-8 and IL-6 (1) the length of bacterial incubation time and cell number could influence extracellular secretion, and (2) extracellular secretion levels differ among epithelial cell type [17]. Taken together, these insinuations would suggest that prolonged and robust respiratory epithelial cell activation attributable to heat-killed Fn cells is able to stimulate an extracellular inflammatory response and, likewise, these inflammatory responses vary among the epithelial cell type. Similarly, in a possible future work, it would be interesting to elucidate the effects of bacterial incubation time and cell count on other inflammation-related signals, especially in terms of gene expression and protein activity.

### 2.2. Heat-Killed Fn Affected the Extracellular Secretion of Inflammatory- and Stress-Related Signals

Sp is a bacterium that has traditionally infected the respiratory system [21], whereas Fn is a bacterium that has mainly occupied the human oral cavity [1,2]. Incubating human respiratory epithelial cells with heat-killed Fn and, subsequently, comparing the results to human respiratory epithelial cells incubated with heat-killed Sp would shed light on the virulence potential of Fn in regard to human respiratory epithelial cells.

**Figure 2:**
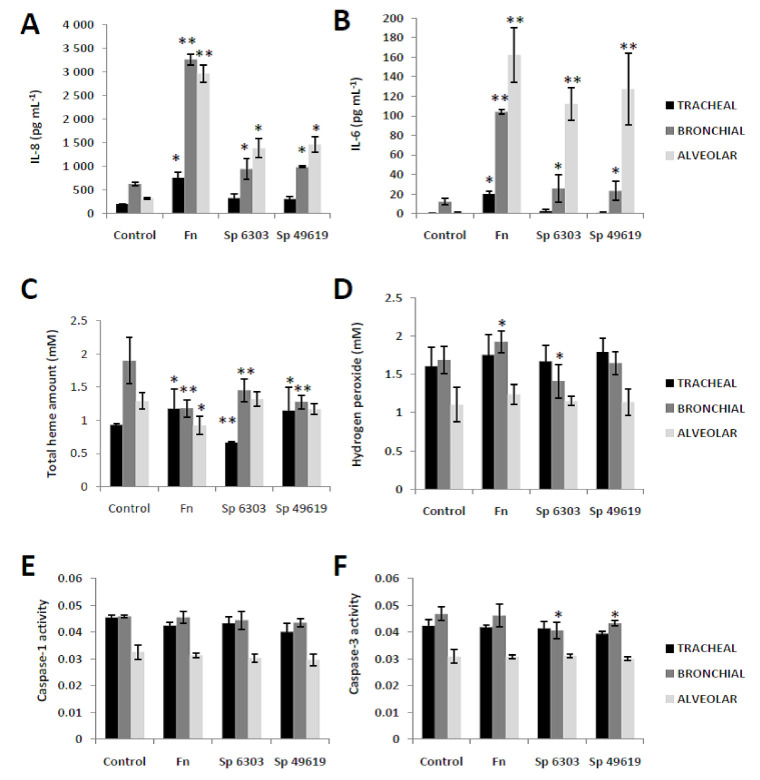
Comparison of inflammatory, stress, and cell death signals secreted from differing primary human respiratory epithelial cells incubated with either heat-killed *Fusobacterium nucleatum* or *Streptococcus pneumoniae*. Assay measurements of secreted inflammatory ((**A**) IL-8 and (**B**) IL-6), stress ((**C**) total heme and (**D**) hydrogen peroxide), and cell death ((**E**) caspase-1 and (**F**) caspase-3)signals are shown. Control (only primary epithelial cells), Fn (primary epithelial cells incubated with *Fusobacterium nucleatum*), and Sp (primary epithelial cells incubated with either *Streptococcuspneumoniae* strain 6303 or 49619) are indicated. The results presented are the mean ± SD utilizing two replicates of three independent samples. Statistical analyses were performed using an Andersen–Darling normality test to check whether the values obtained were normalized (*p*> 0.05) and, if passed, matched-pair *t*-test (* *p* < 0.05; ** *p* < 0.01).

To differentiate the inflammatory response of the varying human respiratory epithelial cells incubated with either heat-killed Fn or Sp, secreted IL-8 and IL-6 were measured. As seen in Figure 2A,B, we found that incubation of heat-killed Fn and Sp (ATCC 6303 and 49619) with human respiratory epithelial cells (tracheal, bronchial, alveolar) resulted in the following: (1) incubation with only heat-killed Fn induced higher IL-8 and IL-6 secretion from human tracheal cells; (2) both human bronchial and alveolar cells incubated with either heat-killed Fn or Sp secreted higher IL-8 and IL-6 amounts; and (3) secreted IL-8 and IL-6 were highest among human respiratory epithelial cells incubated with heat-killed Fn. This would insinuate that the amount of secreted IL-8 and IL-6 depends on the respiratory epithelial cell type and potential bacterial adhesion with either Fn or Sp. Similarly, these results would generally suggest that although heat-killed Fn and Sp were used (which means bacterial activity associated with virulence are inactive), inflammation was nevertheless induced and secreted from the epithelial cells, which would imply that certain signals along the bacterial cell surface initiated the activation and secretion of IL-8 and IL-6 amounts [17].

Heme is a critical component of the inflammatory process [22], which would suggest that elevated levels of secreted IL-8 and IL-6 likewise affected extracellular heme. To establish whether extracellular heme from the varying human respiratory epithelial cells incubated with either heat-killed Fn or Sp are somehow affected, secreted heme were quantified. As shown in Figure 2C, we observed the following: (1) secreted heme differed in all epithelial cell types incubated with heat-killed Fn; (2) secreted heme in both tracheal and bronchial epithelial cells incubated with heat-killed Sp differed from the control; and (3) secreted heme were unchanged among alveolar epithelial cells incubated with heat-killed Sp. Secreted heme could regulate immune response (such as inflammation) as well as serve as a danger signal [23]. Considering that all respiratory epithelial cells varied in secreted heme amounts compared to the control, we believe that secreted heme indirectly affected IL-8 and IL-6 by serving a regulatory role and, likewise, possibly acted as a danger signal, which we think are both dependent on the epithelial cell type [24].

Heme becomes a danger signal either under overwhelming cellular stress or infection [25]. To elucidate whether extracellular oxidative stress-related signals were elevated among the varying human respiratory epithelial cells incubated with either heat-killed Fn or Sp, secreted H_2_O_2_ were determined. As shown in Figure 2D, we found that only secreted H_2_O_2_differed among bronchial epithelial cells incubated with either heat-killed Fn (higher than control) or Sp ATCC 6303 (lower than control). Secreted H_2_O_2_ is attributable to either cell death induction or host defense response and, likewise, is involved in both regeneration and repair [23,26,27]. Based on our results, we surmised that respiratory epithelial cells with either heat-killed Fn or Sp that did not show any change in secreted H_2_O_2_werenot significantly affected by activities associated to cell death, host defense, tissue regeneration nor repair, whereas bronchial epithelial cells with either heat-killed Fn or Sp may have a different set of conditions. IL-6 functions in both inflammation induction and H_2_O_2_ regulation [18] and since secreted IL-6 amounts were elevated in all epithelial cell types incubated with heat-killed Fn and Sp, this could mean that secreted H_2_O_2_ was regulated ascribable to IL-6. Moreover, we suspect that increased amounts of secreted H_2_O_2_ in bronchial epithelial cells incubated with heat-killed Fn are potentially associated with host defense against pathogens (consistent with increased amounts of secreted IL-8 (Figure 2A) and decreased amounts of secreted heme (Figure 2C)), while decreased amounts of secreted H_2_O_2_ in bronchial epithelial cells incubated with heat-killed Sp are probably linked to host protection against injury in order to avoid cell death induction [18].

It was previously reported that caspases can be released from cells [28,29]. To elucidate whether caspases are being secreted from the varying human respiratory epithelial cells incubated with either heat-killed Fn or Sp, secreted CASP1 and CASP3 amounts were quantified. We found that there was no apparent change in secreted CASP1 among the respiratory epithelial cell types regardless of incubation with either heat-killed Fn or Sp (Figure 2E), whereas secreted CASP3 decreased only among bronchial cells incubated with heat-killed Sp (Figure 2F). Secreted CASP1 is a marker of inflammasome activation [30], which is consistent with our observed inflammatory responses (Figure 2A,B), while secreted CASP3 has been linked with bacterial infection, with some pathogens capable of decreasing CASP3 activity [31], as we detected with Sp (Figure 2F). In this regard, we postulate that respiratory epithelial cells incubated with either heat-killed Fn or Sp may not be harmful to the epithelial cell [32] regardless of having an active inflammatory response. Admittedly, additional experimentation is required to further confirm this point.

It is worth mentioning that respiratory epithelial cells incubated with heat-killed Fn induced comparable secretion of most inflammatory- and stress-related signals compared to respiratory epithelial cells incubated with heat-killed Sp. We believe that this would further highlight the virulence potential of Fn in infecting pulmonary tissues [10]. Similarly, considering the potential for heat-killed Fn to stimulate cell death pathways (which, in turn, could suggest a passive secondary mechanism) among the respiratory primary epithelial cells was not fully elucidated, we acknowledge that our current work has limitations since the relationship between cell viability and inflammatory mediator release was not fully established. In this regard and as a possible future work, it would be interesting to establish whether heat-killed Fn could likewise affect the varying cell death-related signals and mechanisms.

### 2.3. Centrality Analysis of Biomolecules Affected by Heat-Killed Fn

**Figure 3:**
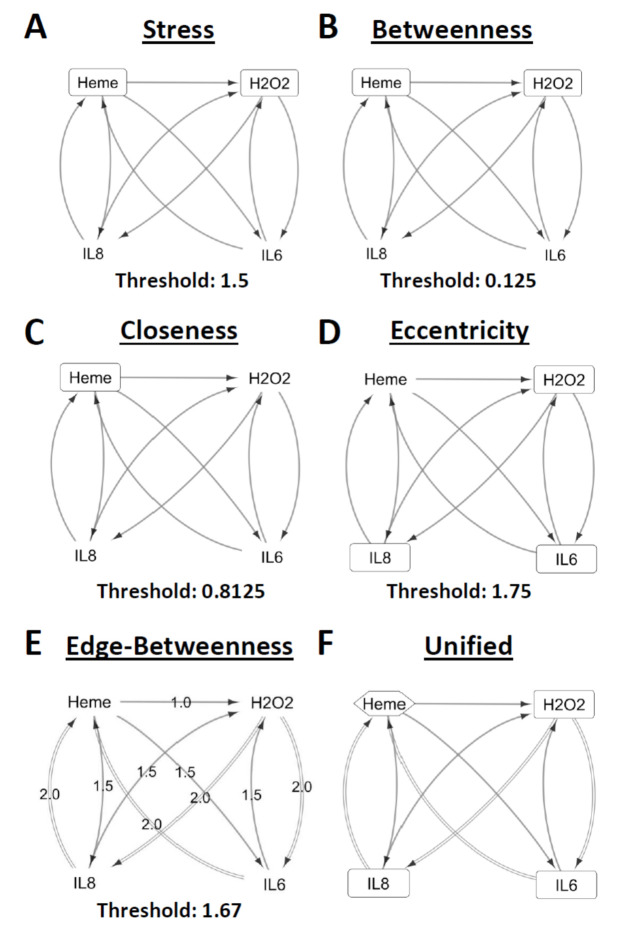
Network design and analysis of secreted inflammatory- and stress-related signals from primary human respiratory epithelial cells incubated with either heat-killed *Fusobacterium nucleatum.* (**A**) stress, (**B**) betweenness, (**C**) closeness, (**D**) eccentricity, and (**E**) edge-betweenness centrality measurements are shown. Threshold for each centrality measurement is indicated below the respective network design. Nodes determined to be above the threshold are indicated in a rectangular box. Edges established to be above the threshold are indicated as double-lined arrows. (**F**) Unified network combines the significant nodes and edges from all centrality measurements. The node considered to important, crucial, and relevant to the network is indicated in a hexagonal box. Nodes that are easily accessed is indicated in a rectangular box.

Centrality analysis allows for the holistic understanding of all network elements found within a network design. In order to elucidate the interconnection between the secreted biomolecules affected by heat-killed Fn (inflammation-related: IL-8, IL-6; stress-related: heme, H_2_O_2_), network design and centrality analyses were performed. It is worth mentioning that since no change was observed among respiratory epithelial cells incubated with heat-killed Fn, both CASP1 and CASP3 were not incorporated in the network design. Based on nodal analyses (stress, betweenness, closeness, and eccentricity centralities), we found that heme was the common node for stress (Figure 3A), betweenness (Figure 3B), and closeness (Figure 3C) centralities while H_2_O_2_ was the common node for stress (Figure 3A), betweenness (Figure 3B), and eccentricity (Figure 3D) centralities. Moreover, both IL-8 and IL-6 nodes were only prominent in eccentricity centrality. These results would insinuate that although both heme and H_2_O_2_ secretion are significantly affected by the presence of heat-killed Fn, only secreted heme has a major role that could putatively affect H_2_O_2_, IL-8, and IL-6 secretion in line with our results (Figure 2A–D) and earlier published works [22,23,27]. Additionally, and based on edge-betweenness centrality analysis, the following interactions were considered significant relative to the overall network designed: H_2_O_2_-IL-8, H_2_O_2_-IL-6, IL-6-heme, and IL-8-heme. This would imply that these interactions were affected by heat-killed Fn’s presence. Furthermore, as seen in the unified network (Figure 3F), we postulate that secreted H_2_O_2_ amounts (in bronchial cells incubated with either heat-killed Fn or Sp) influenced IL-8 and IL-6 secretion which likewise were affected by secreted heme amounts consistent with our results (Figure 2A–D). In regard to tracheal and alveolar cells incubated with either heat-killed Fn or Sp, we similarly believe that secreted IL-8 and IL-6 were affected by secreted heme amounts (Figure 2A–C), which is consistent with the regulatory function of heme in the immune response [23].

## 3. Discussion

Fn is commonly linked to periodontal disease and, likewise, is implicated in various systemic diseases [2,3,4,5,6]. In addition, Fn is considered a commensal bacteria that could eventually turn pathogenic [1,8]. Throughout this study, we attempted to elucidate the virulence potential of heat-killed Fn on primary human respiratory epithelial cells. Considering that bacterial LPS-related respiratory epithelial cell activation and the release of inflammatory responses require serum components not utilized in this study [33], we postulate the involvement of bacterial adhesins.

Bacterial adhesins were initially categorized as adhesive surface structures, which can be classified into bacterial adhesin families (such bacterial pili and curli); however, numerous monomeric surface-bound adhesive proteins have likewise been identified and found to induce signaling events in the host cell that could consequently affect bacterial invasion and inflammatory responses [34]. In general, known fusobacterial adhesins (Aid1, CmpA, Fap2, FomA, RadD) play a role in microbial coaggregation [3,13,35], biofilm formation [12,35], cell death induction [3], bacterial colonization [15], and immune cell inhibition [36]. In this regard and based on our results, we suspect that the extracellular inflammatory response (IL-8 and IL-6 secretion) from the various human epithelial cells is ascribable to Fn adherence (putatively ascribable to fusobacterial adhesins). Moreover, taking into account that bacterial adherence is an important virulence factor [15], we further postulate that a probable Fn adherence may likewise serve a virulence function and that both IL-8 and IL-6 secretion are host responses associated with Fn adhesion to the various human respiratory epithelial cells.

Additionally, considering that secreted heme promotes and exacerbates immune responses [22] while likewise serving a regulatory function with regard to immune response (functioning as a danger signal [23]), we postulate the following: (1) in the case of tracheal cells, an increase in both secreted heme and inflammatory response (secreted IL-8 and IL-6) compared to the control could imply that the stimulation of a regulated inflammatory response was attributable to Fn adhesion which, in turn, similarly functions as a danger signal; and (2) in the case of bronchial and alveolar cells, a decrease in secreted heme while both secreted IL-8 and IL-6 are increased compared to the control would suggest that the regulatory function of secreted heme is decreased, thereby allowing secreted IL-8 and IL-6 levels to drastically increase.

Innate inflammatory responses are mediated by either pathogen-associated molecular patterns (PAMP) or damage-associated molecular patterns (DAMP) and both closely interact during an infection [23,37]. Moreover, release of DAMP components (such as heme) continuously occur (together with PAMP) in the event of a bacterial infection [23]. Considering that the course of infection and disease development along the respiratory track varies [24], we hypothesize that the varying inflammatory- and stress-related responses associated with heat-killed Fn are dependent on the epithelial cell type. In this regard, for tracheal cells incubated with heat-killed Fn, we believe that increases in secreted heme and both secreted IL-8 and IL-6 are consistent with co-stimulating DAMP and PAMP responses which, in turn, induce an inflammatory response (albeit regulated due to the regulatory function of secreted heme) as part of host defense, whereas, for both bronchial and alveolar cells incubated with heat-killed Fn, we suspect that only a PAMP response was stimulated, leading to an inflammatory response as part of host defense, with bronchial cells generating an increase in secreted H_2_O_2_ putatively as an additional host defense component. This would highlight that the mechanism of host defense-linked inflammation response differs in each epithelial cell.

In summary, we believe that in a putative Fn-related disease scenario involving respiratory epithelial cells, whereby infection occurs along the respiratory tract (tracheal to bronchial to alveolar epithelial cells), Fn adherence (possible via fusobacterial adhesin) would serve as a potential virulence factor that would stimulate a host response involving inflammatory signals (secreted IL-8 and IL-6) that differ in each epithelial cell type. More specifically, we postulate the following: (1) along tracheal cells, Fn adherence would trigger increased heme secretion and initiate a regulated inflammatory response; (2) along bronchial cells, Fn adherence would simultaneously initiate an increase in secreted H_2_O_2_ and an inflammatory response ascribable to decreased secreted heme amounts; and (3) along alveolar cells, Fn adherence would instigate the increased secretion of inflammatory responses attributable to a decrease in secreted heme levels. We suspect that, in all three epithelial cells, the differing inflammatory response mechanisms are related to host defense. Similarly, regardless of the epithelial cell-specific inflammatory mechanism, these are putatively not harmful. Taken together, we propose that any potential Fn-driven inflammation along the respiratory track would be initiated by differing epithelial cell-specific inflammatory mechanisms that are collectively dependent on secreted heme.

## 4. Materials and Methods

### 4.1. Bacterial Cell Culture and Heat Inactivation

*F. nucleatum* ATCC 25586 and two different strains of *Streptococcus pneumonia* (ATCC 6303 and ATCC 49619) were used for this study. For the purpose of this study, *F. nucleatum* will be indicated as Fn while *S. pneumonia* will be indicated as Sp. Fn were cultured in BHIB supplemented with 5 μg mL^−1^ hemin and 0.5 μg mL^−1^ menadione while both strains of *S. pneumonia* (Sp) were cultured in brain heart infusion broth (BHIB; Becton, Dickinson and Company, Sparks, MD, USA). Both bacterial cell cultures were grown under an anerobic condition of 10% H_2_, 10% CO_2_, and 80% N_2_ with incubation set at 37 °C for 24–72 h. Intact bacterial cells were obtained from the supernatant via centrifugation at 7000× *g* for 10 min at 4 °C and, subsequently, passed through a 0.22-μm pore filter membrane before being resuspended in appropriate human respiratory epithelial cell culture medium depending on downstream application. Bacterial cell density was adjusted to 1.0 × 10^8^ CFUmL^−1^ and eventually heat-killed at 60 °C for 1h before being stored at −80 °C until use in downstream experimentation. Considering Fn is an anaerobic bacteria, thus, unable to survive along the respiratory tract, we used heat-killed Fn and, moreover, heat-killed Fn was used since we wanted to focus on the possible effects of Fn attachment (possibly attributable to fusobacterial adhesins along the bacterial surface) on inducing inflammation, which, in turn, may explain why Fn is considered a risk factor for certain respiratory diseases [9,10].

### 4.2. Primary Human Epithelial Cell Culture and Treatment

Primary human tracheal, bronchial, and alveolar epithelial cells were cultured in an appropriate medium following the manufacturer’s recommendations. More specifically, Bronchial Epithelial Cell Medium was used to culture both tracheal and bronchial epithelial cells, while Alveolar Epithelial Cell Medium was used to culture alveolar epithelial cells. Both primary cells and growth medium were purchased from ScienCell Research Laboratories (Carlsbad, CA, USA). Briefly, epithelial cells were incubated at 37 °C prior to treatment. Cell growth and concentration were visually confirmed through a microscope and, likewise, via optical density. Sample treatment involves incubating a heat-killed bacterial cell (either Fn or Sp) with an epithelial cell at a final concentration of either 1.0 × 10^7^ CFU mL^−1^ or 1.0 × 10^8^ CFU mL^−1^ (depending on downstream experimentation). Both bacteria and epithelial cell culture supernatants from all treatments were isolated and stored at −80 °C until use in downstream experimentation.

### 4.3. Measurement of Secreted Inflammation- and Stress-Related Signals

Secreted inflammation-related signals quantified include IL-8 and IL-6 cytokines, whereas secreted stress-related signals measured include total heme and hydrogen peroxide (H_2_O_2_). Additionally, caspase-1 (CASP1) and caspase-3 (CASP3) were likewise quantified to elucidate whether detected inflammation- and stress-related signals resulted in cell death. Pierce^®^ Microplate BCA Protein Assay Kit-Reducing Agent Compatible Kit (Thermo Scientific, Carlsbad, CA, USA) was used to standardize cell-free supernatant prior to downstream analyses. Secreted IL-8 and IL-6 amounts in the cell-free supernatants were measured using commercially available IL-8 and IL-6 ELISA kits (R&D Systems, Minneapolis, MN, USA). QuantiChrom^TM^ Heme Assay Kit (BioAssay Systems, Hayward, CA, USA) was used to measure secreted heme (free heme and heme-proteins). Red Hydrogen Peroxide Assay Kit (Enzo Life Sciences, Plymouth Meeting, Pennsylvania, PA, USA) was used to measure secreted H_2_O_2_. Secreted CASP1 and CASP3 were quantified usingCaspase-1/ICE and Caspase-3/CPP32 Colorimetric Assay Kits (Biovision, Milpitas, CA, USA), respectively. All kits were used following the manufacturer’s recommendations.

### 4.4. Network Design and Analysis

Network analysis utilizes a computer-based modelling environment and has been developed to understand the relationship of various biochemical signals involved in a given process [38]. We used Cytoscape to understand the network relationship of the varying secreted signals studied [39]. For this study, a network design of the putative interrelationship between the various signals studied was initially made and, more importantly, we measured the following topological centralities: (1) stress centrality, to determine the importance of each component with regard to the whole network; (2) betweenness centrality, to know which signals are considered crucial to the whole network studied; (3) closeness centrality, to establish the signals that are relevant to the whole network; (4) eccentricity centrality, to determine which signals are readily accessible with regard to the other signals and the whole network; and (5) edge betweenness centrality, to elucidate the important interaction between the two signals [40]. Briefly, the threshold for each centrality was first computed and, eventually, centrality values above the computed threshold would be considered significant edges and nodes.

### 4.5. Statistical Analyses

All experiments were performed using three independent samples (*n* = 3) and data are presented as mean ± standard deviation. An Andersen–Darling normality test was first performed to check whether the values obtained were normalized (*p* > 0.05). The statistical significance of differences was further elucidated using matched-pair *t*-test, wherein a significance level of 95% (*p* < 0.05) was considered statistically significant.

### 4.6. Statement on Reproducibility

The consilience of the results obtained is inline with result reproducibility and, more importantly, the consistency of the results obtained with previous works aligns with inferential reproducibility [41,42].
